# Patient-specific prediction of SEEG electrode bending for stereotactic neurosurgical planning

**DOI:** 10.1007/s11548-021-02347-8

**Published:** 2021-03-24

**Authors:** Alejandro Granados, Yuxuan Han, Oeslle Lucena, Vejay Vakharia, Roman Rodionov, Sjoerd B. Vos, Anna Miserocchi, Andrew W. McEvoy, John S. Duncan, Rachel Sparks, Sébastien Ourselin

**Affiliations:** 1 School of Biomedical Engineering and Imaging Sciences, King’s College London, London, UK; 2 National Hospital for Neurology and Neurosurgery, London, UK

**Keywords:** Surgical planning, SEEG, Prediction of trajectory

## Abstract

**Purpose:**

Electrode bending observed after stereotactic interventions is typically not accounted for in either computer-assisted planning algorithms, where straight trajectories are assumed, or in quality assessment, where only metrics related to entry and target points are reported. Our aim is to provide a fully automated and validated pipeline for the prediction of stereo-electroencephalography (SEEG) electrode bending.

**Methods:**

We transform electrodes of 86 cases into a common space and compare features-based and image-based neural networks on their ability to regress local displacement ($\mathbf{lu}$) or electrode bending ($\hat{\mathbf{eb }}$). Electrodes were stratified into six groups based on brain structures at the entry and target point. Models, both with and without Monte Carlo (MC) dropout, were trained and validated using tenfold cross-validation.

**Results:**

mage-based models outperformed features-based models for all groups, and models that predicted $\mathbf{lu}$ performed better than for $\hat{\mathbf{eb }}$. Image-based model prediction with MC dropout resulted in lower mean squared error (MSE) with improvements up to 12.9% ($\mathbf{lu}$) and 39.9% ($\hat{\mathbf{eb }}$), compared to no dropout. Using an image of brain tissue types (cortex, white and deep grey matter) resulted in similar, and sometimes better performance, compared to using a T1-weighted MRI when predicting $\mathbf{lu}$. When inferring trajectories of image-based models (brain tissue types), 86.9% of trajectories had an MSE$\le 1$ mm.

**Conclusion:**

An image-based approach regressing local displacement with an image of brain tissue types resulted in more accurate electrode bending predictions compared to other approaches, inputs, and outputs. Future work will investigate the integration of electrode bending into planning and quality assessment algorithms.

**Supplementary Information:**

The online version supplementary material available at 10.1007/s11548-021-02347-8.

## Introduction

Stereo-electroencephalography (SEEG) is used to aid in the localisation of the epileptogenic zone (EZ) in patients with drug-refractory focal epilepsy [14]. SEEG typically requires the placement of 10 to 16 depth electrodes to record electrophysiological brain activity from specific target regions. Precise placement of electrodes is crucial since SEEG is used to locate the EZ in patients with discordant non-invasive anatomo-electro-clinical investigations or where no pathology is present on MRI [2]. Electrode implantation errors can lead to missing onset events due to the limited spatial sampling of electrodes [14], and an increase in risk complications, such as haemorrhage [13, 18].

To place electrodes, stereotactic neurosurgical techniques rely on patient-specific imaging in combination with either a frame, frameless system or robotic device to accurately drill a small burr hole on the skull through which an electrode is inserted to reach a target in the brain. Surgical planning for these procedures consists of specifying an entry point, drilling angle to the skull, and implantation depth for each electrode whilst avoiding critical structures [17]. These parameters may be optimised by computer-assisted planning algorithms and together describe the intended target. Electrode implantation accuracy is assessed post-operatively from a computerised tomography (CT) image co-registered to MRI. Quality assessment is mostly related to angle and distance (Euclidean or lateral shift) metrics of entry point and target point between implanted and planned trajectories [8]. Even if the burr hole is accurately placed and oriented, and the electrode is correctly inserted, deviations from planned trajectories may occur due to the surgical technique used, the structural and biomechanical properties of soft tissue interacting with the tool, and the mechanical properties of electrodes [8]. Electrode bending is typically not accounted for in either planning algorithms, where straight trajectories are assumed, or in quality assessment, where typically only metrics related to entry and target points are reported.

In previous work, we modelled electrodes as elastic rods where orthogonal material frames were computed between interpolated points comprising the trajectory. The rate of change between two consecutive frames, i.e. local bending (Darboux vectors), was used as three degrees-of-freedom (dof) labels for regression from handcrafted features using random forests, a feed-forward neural network, and long short-term memory gates [7]. This approach was later extended to regress 3-dof local displacement since it is more clinically relevant [6]. In that work, we proposed a neural network directly or with Monte Carlo (MC) dropout to quantify epistemic uncertainty (a measure of the inability to ascertain the validity of the chosen model and related parameters) in the prediction. Whilst these models framed electrode bending as a data-driven task, it remains unclear what the best output label to regress is. For instance, electrode bending could be characterised by a displacement offset from a rigid trajectory (i.e. local displacement $\mathbf{lu}$), or by a vector indicating the direction the electrode may deviate at that point in space (i.e. electrode bending $\hat{\mathbf{eb }}$). Moreover, further investigation is necessary to evaluate a more general or streamlined approach to learn bending by using information directly from medical images rather than handcrafted features along electrode trajectories.

The aim of this work is to: 1) assess two data-driven approaches for predicting implanted electrode trajectories using a total of 96 handcrafted features or using electrode direction and a 3D image and 2) validate the predictive capabilities of both approaches when regressing either local displacement or an electrode bending direction in 86 cases consisting of a total of 852 electrodes.

## Novel contribution

Our main motivation is to predict electrode bending in patient-specific SEEG electrode implantations. The main contributions of this work are: 1) the design of a streamlined approach to overcome the limitations of approaches using handcrafted features, 2) an investigation of the predicting capabilities of regressing local displacement versus electrode bending direction, and 3) a fully automated pipeline for visualisation and predicting SEEG electrode bending that is publicly available to the scientific community.^1^

## Methods

### Data

Pre-operative T1-weighted MR (T1w) and post-operative CT images of 86 refractory epilepsy SEEG implantation cases comprising a total of 852 electrodes were acquired at the National Hospital for Neurology and Neurosurgery (Queen Square, London, UK) over a period of 5 years (2015-2020). The electrodes were implanted by the same neurosurgeon as specified by the plan using a frameless system. The surgical technique has improved over time with some cases implanted manually and others with the use of a computer-assisted planning algorithm [17], using a rigid stylet short from target point [16], and most recently 16 cases using a robotic system [3]. T1w and CT images are co-registered with a rigid transformation using NiftyReg (v1.5.43) [15]. From the T1w, we obtained a parcellation of the brain anatomy using Geodesic Information Flow (GIF) via NiftyWeb (GIF v3.0) [1]. An image containing masks, referred as CWD, corresponding to (c)ortex, (w)hite, and (d)eep grey matter was generated from the parcellation. Smoothed 3D surface meshes of the scalp, cortex, white, and deep grey matter were generated from the CWD [7]. The position of the electrode contacts and bolts (Ad-Tech Med Instr Corp, USA) was identified automatically as described in [9]. Electrode trajectories were interpolated at 1 mm intervals using a shape-preserving piece-wise cubic Hermite interpolation (PCHIP). For a trajectory defined by entry, target, and contacts points, *I* interpolated points are created 1 mm apart with positions $\mathbf{x} _i$, where $\mathbf{x} _{I-1}$ is the target point. In this work, rather than using a planned trajectory to characterise an electrode that is not subject to bending, we use a *rigid trajectory*, which is defined as a straight line in the direction of the bolt. Rigid trajectories are similarly interpolated at 1 mm intervals. Electrodes were stratified into six groups based on brain structures of the entry and target point: superior frontal gyrus (***sfg***), middle frontal gyrus (***mfg***), inferior frontal orbital gyrus (***ifog***), temporal gyrus (***tg***), anterior/posterior cingulate gyrus (***apcg***), and parietal/occipital lobes (***po***) (**Supplemental Material (SM)** Table 1).

**Fig. 1:**
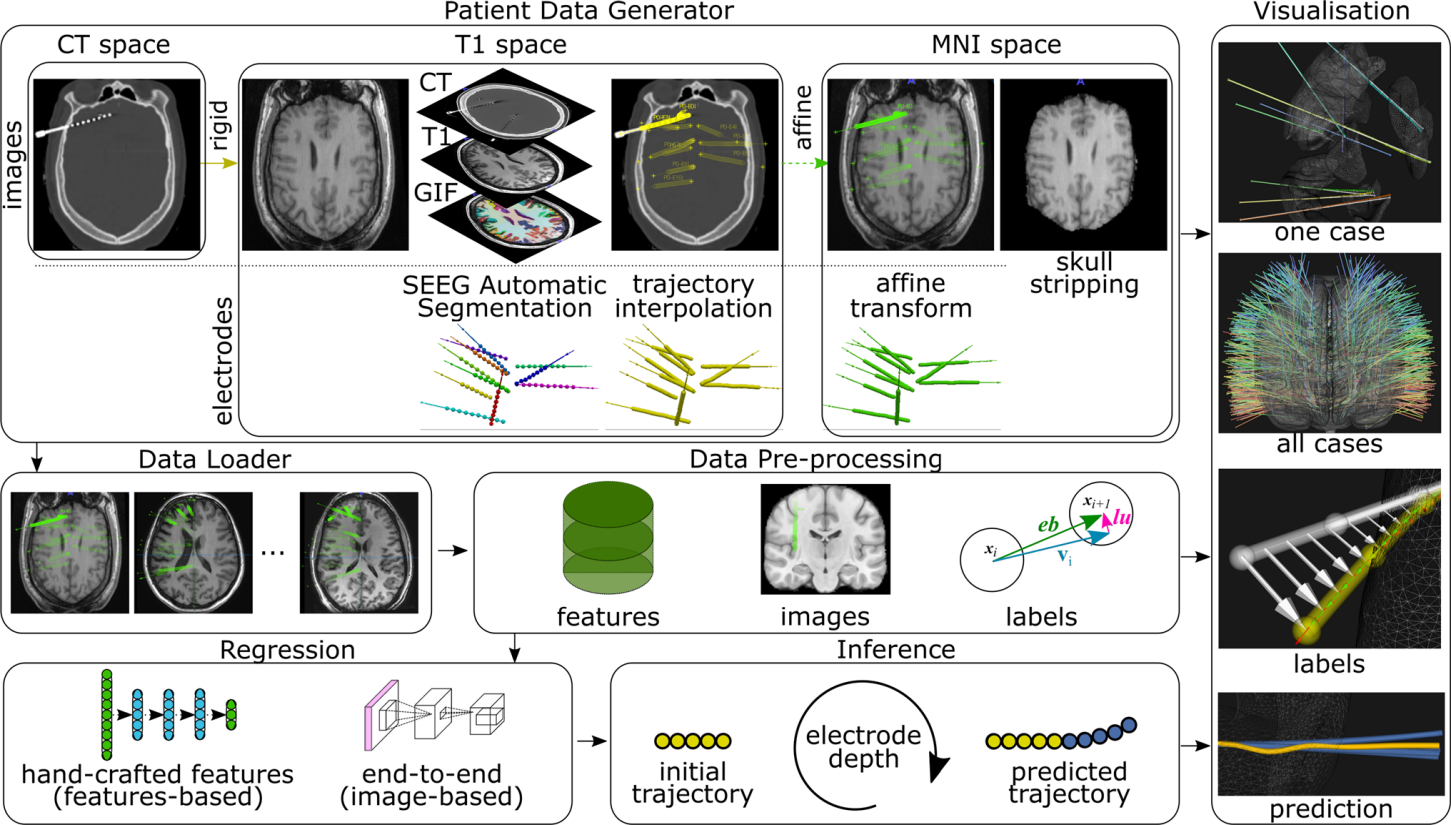
Pipeline. SEEG electrodes are identified automatically from co-registered pre-operative T1w, parcellation (GIF), and post-operative CT images in the T1w space. Electrode trajectories are interpolated at 1 mm intervals and then registered into MNI space via an affine transformation. Electrodes in the MNI space are loaded into memory and pre-processed to generate input data and labels for training both regression models. To infer electrode trajectories, a trained model is iteratively used to predict the next interpolation point from an initial trajectory based on electrode bolt direction. Visualisation is used to render electrodes of a patient, of all cases, and of predictions in relation to anatomy

### Pipeline

We transform rigid and implanted electrode trajectories into the Montreal Neurological Institute (MNI) space and compute inputs and labels in that space as follows. We first register with an affine transformation the patient-specific T1w to the MNI152 template T1w [4] using NiftyReg [15]. The computed affine transformation is then applied to electrode trajectories and surface meshes, aligning these objects in MNI space (Fig. 1). We normalise ([0,1]) and skull-stripped the transformed T1w using ROBEX (v1.2) [11].

**Fig. 2:**
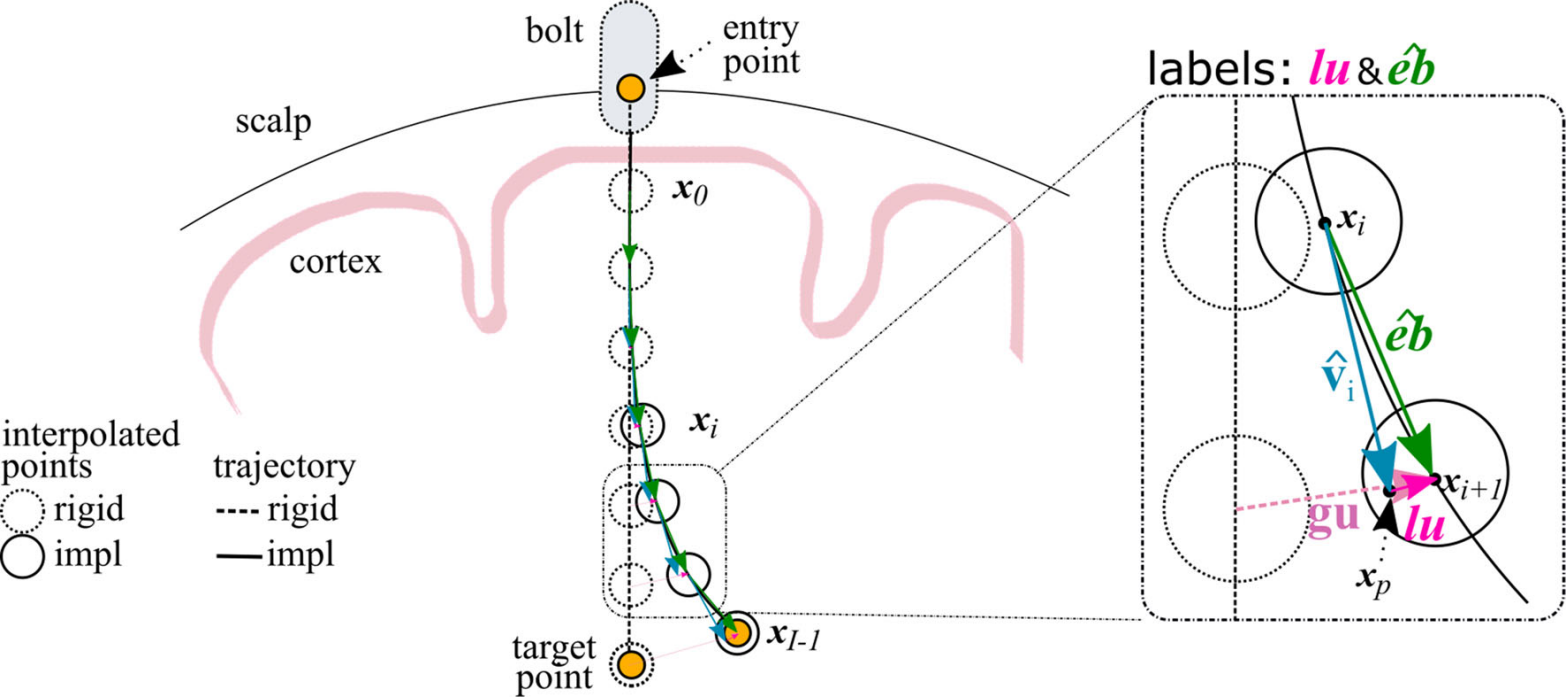
Schematic definition of an implanted electrode (solid lines) that deviated from a rigid trajectory (dashed lines). **Trajectories** are interpolated at 1 mm intervals, and the deviation at each interpolated point $\mathbf{x} _i$ from rigid trajectory is defined as global displacement $\mathbf{gu}$. Electrode bending is characterised either by local displacement $\mathbf{lu}$ plus electrode direction $\hat{\mathbf{v }}_i$ or by a unit vector $\hat{\mathbf{eb }}$ between interpolated points $\mathbf{x} _i$ and $\mathbf{x} _{i+1}$

### Definition of labels for regression

We compute two types of labels that characterise electrode bending at trajectory points $x_i$: a) local displacement $\mathbf{lu}$ and b) electrode bending $\hat{\mathbf{eb }}$. Local displacement $\mathbf{lu}$ (pink arrow in Fig. 2; Eq. 1) is a 3-dof vector that measures the offset (in mm) from a projected point $\mathbf{x} _p$ located 1 mm away from an interpolated point $\mathbf{x} _i$ in the direction of the electrode trajectory $\hat{\mathbf{v }}_i$. Electrode bending direction $\hat{\mathbf{eb }}$ (green arrow in Fig. 2; Eq. 2) is a 3-dof unit vector with origin at $x_i$ and pointing towards $x_{i+1}$. A new predicted point $\tilde{\mathbf{x }}_{i+1}$ can then be found via either Eq. 1 or Eq. 2 depending on if local displacement or bending direction is defined. Note that the formulations are equivalent as $\hat{\mathbf{v }}_i+\mathbf{lu} = \hat{\mathbf{eb }}$. For the model architecture and inputs described in Sec. 3.4.1, we regress one of these labels.

1 $$\begin{aligned} \hat{\mathbf{v }}_i=\frac{\mathbf{x }_i-\mathbf{x }_{i-1}}{|\mathbf{x }_i-\mathbf{x }_{i-1}|}\nonumber \\ \mathbf{x }_{i+1}=\mathbf{x }_i+1*\hat{\mathbf{v }}_i+\mathbf{lu} {\text {, where}}\nonumber \\ \mathbf{x }_p=\mathbf{x }_i+1*\hat{\mathbf{v }}_i ~{\text {and}}~ \mathbf{lu =\mathbf{x }_{i+1}-\mathbf{x }_p} \end{aligned}$$

2 $$\begin{aligned} \hat{\mathbf{eb }}=\frac{\mathbf{x }_{i+1}-\mathbf{x }_{i}}{|\mathbf{x }_{i+1}-\mathbf{x }_{i}|}\nonumber \\ \mathbf{x }_{i+1}=\mathbf{x }_i+1*\hat{\mathbf{eb }} \end{aligned}$$

### Machine learning algorithms

#### Models

**Fig. 3:**
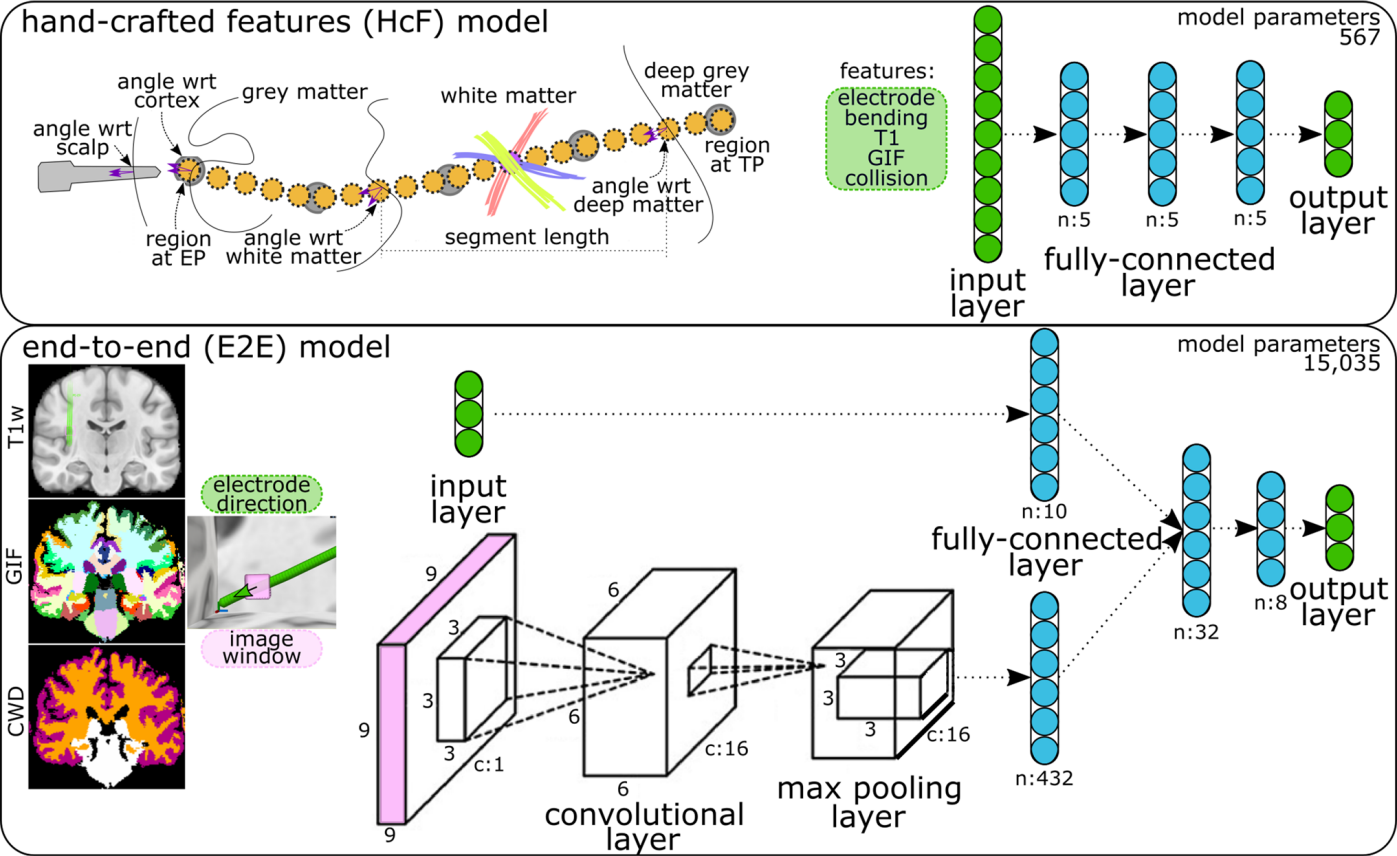
Architecture of machine learning algorithms. **HcF:** A total of 96 features are generated at each interpolated point along electrode trajectory and used as inputs to three hidden blocks (each with linear, PReLU, and dropout layers). **E2E:** Electrode direction and a $9\times 9\times 9$ image window (either from T1w, GIF or CWD) are used as inputs to a linear and a convolutional/max pooling layer, respectively. The resulting layers are stacked together and pass through two fully connected layers (each with linear, LeakyReLU, batch normalisation, and dropout layers)

Handcrafted features (HcF). Similar to previous work [6, 7], we computed features related to electrode implantation, bending, structure, and collision at each interpolated point (**SM** Table 2). We normalised continuous variables and encode categorical variables into one-hot encoding vectors including when a stylet was used (yes, no, no information available), type of anatomical region (cortex, white, deep matter), entry points by lobe (frontal, central, temporal, parietal, occipital), and target points by region (frontal, central, temporal, parietal, occipital, insula, cingulum). In total, there are 96 features used as inputs to a neural network model composed of a parametric rectified linear unit (PReLU) activation, followed by three hidden blocks, and a fully connected layer (Fig. 3 top). Each hidden block consists of a fully connected layer with five neurons, a PReLU activation layer, and a dropout layer.

End-to-end (E2E). We use a multiple-input model architecture where inputs are comprised of the 3-dof electrode direction $\hat{\mathbf{v _i}}$ (Eq. 1) and a one-channel $9\times 9\times 9$ image window at interpolated points (Fig. 3 bottom). The window size is specified such that it is large enough to capture local information along the trajectory, whilst small enough to run the convolutional and max pooling layers. We study windows constructed from T1w, GIF, and CWD images. The electrode direction $\hat{\mathbf{v _i}}$ input passes through a fully connected layer with 5 neurons followed by a rectified linear unit (ReLU) activation. The image window input passes through a 3D convolutional layer with a kernel size of $3\times 3\times 3$ and zero padding, followed by a leaky ReLU activation unit, and max pooling of size $2\times 2\times 2$. These two input layers are stacked into two sequential blocks consisting of a fully connected layer followed by a leaky ReLU activation unit, a 1D batch normalisation layer, and a dropout layer. The first block consists of 32 neurons, whilst the second consists of 8 neurons. A final fully connected layer with 3 neurons is used to infer the outputs.

#### Training

The proposed algorithms were trained using the Adam optimiser with a weight decay of $10^{-3}$ to minimise the mean squared error (MSE) loss function ${\mathcal {L}} = \frac{1}{N} \sum _{n=1}^{N} (\mathbf{y} _n - \hat{\mathbf{y }}_n)^2$, where $\mathbf{y} _n$ is the ground truth label ($\mathbf{lu}$, or $\hat{\mathbf{eb }}$), and $\tilde{\mathbf{y }}_n$ is the inferred output, corresponding to the predicted bending of the *n*th point along the trajectory of an electrode with a total of N points. Models were trained for 200 epochs with validation performed every 5 epochs. We dynamically reduced the learning rate $lr=10^{-3}$ by a factor of 10 when the training epoch loss stopped improving (i.e. on a plateau) using a scheduler.

#### Inference

Regardless of the label regressed during inference, predicted trajectories are computed as follows. First, an initial trajectory of 5 points (5 mm) is extracted from a rigid trajectory in the direction of the bolt. Then, we iteratively extend the tip of the electrode by one point (1 mm) up to a specified depth. At each iteration, we generate inputs at the position of the last interpolated point, regress either $\mathbf{lu}$ (Eq. 1) or $\hat{\mathbf{eb }}$ (Eq. 2), and compute the next point $\mathbf{x} _{i+1}$. At inference time, we used the dropout layers to build stochastic models where the outputs are described as a probability distribution, which is approximately equivalent to having probability distributions of the model weights. This allows for variance in HcF and E2E model predictions to be computed. In particular, epistemic uncertainty can be computed as a Bayesian approximation to a Gaussian process, using MC dropout [5]. This is achieved by computing *T* random forward passes which are averaged to compute the final prediction. We use dropout probability of 0.1 and 200 forward passes.

#### Experiment design and validation

We study the predicting performance of $\mathbf{lu}$ and $\hat{\mathbf{eb }}$ in HcF and E2E models reporting performance across anatomical groups (**SM** Table 1). We evaluate the benefit of quantifying uncertainty and study three distinct types of inputs (T1w, GIF, CWD) for the E2E approach. A model is trained for each anatomical group with tenfold cross-validation, where we first randomly selected 10% of the cases (8 cases; 86 electrodes) as a hold-out test set and then split the remaining cases into tenfold, 9 for training (70 cases) and 1 for validation (8 cases). We then run inference on the hold-out test set to compute predicted trajectories and report MSE of these trajectories against the ground truth for the best performing models. In the remaining of the manuscript, we denote $MSE_t$ to refer to the MSE of the model cross-validation, and $MSE_i$ to refer to the MSE of predicted trajectories. Note that small values are expected for $MSE_t$ since $\mathbf{lu}$ refers to a local displacement (see vector in pink in Fig. 2, close-up view), whereas values in the order of millimetres are expected for $MSE_i$, since these relate to the actual trajectories and are a better indicator of performance for our proposed approach.

**Fig. 4:**
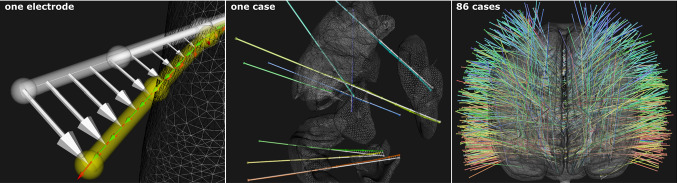
Visualisation of the SEEG electrode trajectories dataset used in our study. Smoothed surface meshes (wireframe mesh) generated from parcellation of relevant entry and target points of implanted electrodes. For each electrode, rigid trajectories (translucent white tube), implanted trajectories (translucent coloured tube - note that colours are randomly allocated), and contacts (spheres) are rendered in 3D. We also render vector fields at interpolated points along the trajectory, including electrode direction $\hat{\mathbf{eb }}$ (green arrows), local displacement $\mathbf{lu}$ (as a unit vector for visualisation in red arrows), and global displacement $\mathbf{gu}$ (white arrows). We illustrate electrode trajectories for one case (centre) and for the entire dataset consisting of 86 cases (right)

### Implementation

The proposed system is implemented in Python (v3.7). Medical images are processed in SITK (v1.2.4), and visualisation of electrodes and surface meshes is done in VTK (v9.0.1). Machine learning algorithms are implemented in PyTorch (v1.5.1) and trained on a Nvidia DGX cluster using Docker containers.

## Results

### Data

Electrode trajectories transformed into MNI space are shown in Fig. 4 and in **SM** Fig. 2 across groups. The left image of Fig. 4 illustrates, for one implanted electrode (yellow), the $\mathbf{lu}$ (red) and $\hat{\mathbf{eb }}$ (green) labels. Note that $\mathbf{lu}$ has been normalised (unit vector) to aid in its visualisation. Rigid trajectories (white) are displayed with a corresponding vector field (white arrows) indicating the amount of global displacement $\mathbf{gu}$ at interpolated points. Global displacement $\mathbf{gu}$ is shown in **SM** Fig. 1 across anatomical regions.

### Model validation

Results of the tenfold cross-validation for $\mathbf{lu}$ and $\hat{\mathbf{eb }}$ labels are shown in Table 1. Values indicate the mean and standard deviation (in $10^{-3}$ mm) of $MSE_t$ across folds for the hold-out test set. Notice that $MSE_t$ errors are lower for $\mathbf{lu}$ than $\hat{\mathbf{eb }}$ labels since local displacement is the distance from a projected point along the electrode direction, whilst $\hat{\mathbf{eb }}$ is a unit vector representing the direction of bending. E2E models trained faster than HcF models when regressing $\hat{\mathbf{eb }}$, although no substantial time differences were observed between models when regressing *lu* (**SM** Table 3).

HcF vs E2E. The results in Table 1 indicate that the predictive performance of the streamlined end-to-end (E2E) approach that uses only electrode direction and a 3D image window outperformed the handcrafted (HcF) approach comprising 96 features. Improvements are particularly observed in the ***apcg*** group followed by ***sfg***, ***mfg***, ***ifog***, and ***po*** groups for $\mathbf{lu}$.

E2E image window. T1w and CWD image inputs resulted in better predictions compared to GIF. For $\mathbf{lu}$, CWD performed similarly, and sometimes better, than T1w in ***sfg***, ***mfg***, ***ifog***, and ***tg*** groups. However, performance for CWD was twice as worst in ***apcg***, and ***po*** groups. For $\hat{\mathbf{eb }}$, models using T1w image inputs outperformed CWD in all groups except ***apcg***.

Stochastic models. Dropout in neural networks, which consists of randomly setting elements of the inputs to zero, is used as a way to avoid overfitting and is mathematically equivalent to an approximation to a Gaussian Process probabilistic model [5]. We use MC dropout to compute the predictive mean and uncertainty by collecting results of stochastic forward passes. This enables us to model how the estimation of the neural network parameters affects the inference step. In this work, models using MC dropout outperformed models that directly predicted the outputs. Improvements were negligible in HcF models. In E2E models, we observe improvements of $\approx 7$% when using GIF as input for both $\mathbf{lu}$ and $\hat{\mathbf{eb }}$ labels, $\approx 11$% when using T1w or CWD inputs in $\mathbf{lu}$ labels, and 28% or 40% of $\hat{\mathbf{eb }}$ models when using CWD or T1w, respectively.

**Table 1:** Tenfold cross-validation of HcF and E2E models regressing $\mathbf{lu}$ (in $10^{-3}$ mm) and $\hat{\mathbf{eb }}$ (normalised to unit length; in $10^{-3}$ mm). Mean and standard deviation of $MSE_t$ across folds for a direct and a stochastic (MC dropout) model are reported. We evaluate the performance of the E2E model with different inputs: T1w, GIF, and CWD

	**Model**	**HcF**		**E2E**					
	*Experiment*	*N/A*		*T1w*		*GIF*		*CWD*	
**Label**	MC dropout	No	Yes	No	Yes	No	Yes	No	Yes
**lu**	sfg	3.09 (0.47)	3.08 (0.48)	0.43 (1.09)	0.35 (1.02)	0.36 (1.03)	0.35 (1.02)	**0.23** (0.64)	**0.20** (0.57)
	mfg	4.26 (0.98)	4.28 (0.97)	0.43 (1.01)	0.41 (0.98)	0.70 (1.85)	0.70 (1.84)	0.43 (1.03)	0.41 (0.99)
	ifog	1.16 (0.30)	1.12 (0.23)	**0.13** (0.35)	0.11 (0.30)	0.44 (1.20)	0.40 (1.17)	0.14 (0.38)	**0.10** (0.30)
	tg	2.03 (1.31)	1.99 (1.32)	0.46 (1.36)	0.46 (1.35)	0.47 (1.37)	0.47 (1.37)	**0.15** (0.39)	**0.13** (0.35)
	apcg	3.78 (0.85)	3.78 (0.83)	**0.14** (0.36)	**0.11** (0.29)	0.64 (1.51)	0.53 (1.50)	0.22 (0.61)	0.18 (0.49)
	po	3.23 (0.33)	3.26 (0.32)	**0.32** (0.88)	**0.31** (0.86)	0.65 (1.73)	0.60 (1.73)	0.62 (1.78)	0.62 (1.78)
$\hat{\mathbf{eb }}$	sfg	62.50 (42.57)	62.30 (43.13)	**15.98** (7.31)	**8.14** (7.98)	87.79 (42.00)	80.73 (44.00)	21.14 (8.76)	14.78 (9.33)
	mfg	56.38 (11.01)	56.35 (12.22)	**26.92** (4.75)	**16.30** (2.50)	71.70 (59.74)	60.26 (62.74)	27.34 (4.61)	17.35 (3.36)
	ifog	41.96 (5.05)	42.08 (7.36)	**25.66** (9.58)	**14.08** (8.35)	185.58 (123.5)	175.60 (131.65)	54.07 (80.68)	44.72 (83.61)
	tg	89.42 (93.12)	87.39 (95.67)	**24.49** (7.52)	**15.69** (7.19)	88.12 (98.00)	79.45 (100.13)	56.23 (82.55)	48.07 (84.31)
	apcg	72.10 (49.48)	72.40 (51.52)	29.22 (20.65)	17.70 (20.13)	143.69 (82.28)	137.87 (86.25)	**24.87** (3.59)	**13.39** (2.24)
	po	73.83 (5.64)	71.70 (6.21)	**36.31** (15.01)	**25.30** (16.44)	186.13 (91.58)	178.95 (96.09)	62.5 (41.5)	48.42 (34.80)

### Trajectory validation

**Fig. 5:**
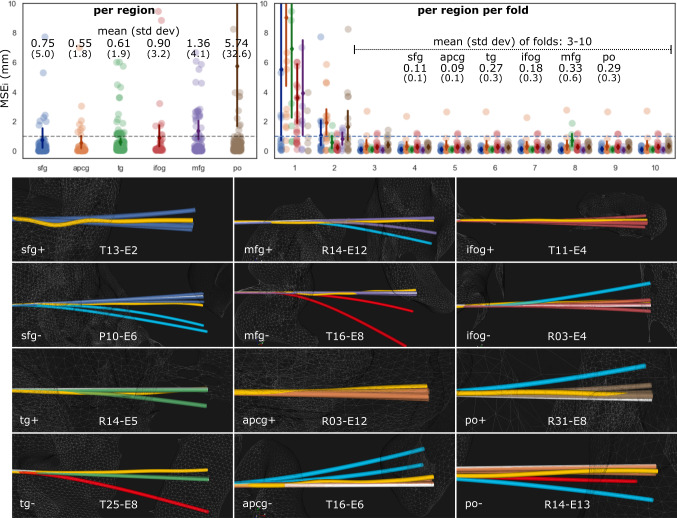
Electrode trajectory prediction of **lu** using E2E CWD model. *Top:* Strip plots of $MSE_i$ between implanted and predicted trajectories across regions (left), and across folds (right). Notice that these colours are used to plot inferred trajectories in the figures below. Mean (standard deviation) is shown for each group and across folds 3 to 10. *Bottom:* Examples of inferred trajectories (case-electrode) of all folds across groups for good (+) and difficult (-) cases. Implanted (yellow—ground truth) and rigid (white) trajectories are shown. Inferred trajectories are coloured: a) according to the anatomical group (see top-left strip plot) when predictions are within $MSE_i$=1 mm, b) in light blue when predictions overestimate bending (i.e. bending is predicted in the same direction in relation to the ground truth but is overestimated), and c) in red when predictions result in the wrong direction in relation to the ground truth

$MSE_i$ computed between predicted and implanted trajectories of 860 trajectories was below 1 mm for 208 electrodes (24.2%) when predicting $\hat{\mathbf{eb }}$ using T1w as input. This increased to 708 (82.3%), or 747 (86.9%) when predicting $\mathbf{lu}$ using T1w or CWD as input, respectively. For the best performing model, E2E CWD **lu**, $MSE_i$ is plotted in Fig. 5 (top) across regions and folds, along a horizontal (dashed) line indicating the 1 mm threshold. On average, ***apcg*** was the group with the lowest $MSE_i$, whereas electrodes predicted for the group ***po*** had the highest errors, followed by ***mfg***. Note that the first fold was the least accurate, followed by the second fold, whereas the $MSE_i$ shows the remaining folds are compared to the MSE of rigid vs implanted trajectories for ***sfg***: 0.1(0.1), ***apcg***: 0.09(0.1), ***tg***: 0.27(0.3), ***ifog***: 0.1(0.05), ***mfg***: 0.34(0.7), and ***po***: 0.25(0.19). We found a negligible positive correlation between electrode length and $MSE_i$ across anatomical groups (SM Fig. 3). In Fig. 5 (bottom), we illustrate how predicted electrode trajectories sometimes bend correctly in the interface between grey matter and white matter, but then do not predict bending occurring deeper.

## Discussion

In contrast to previous work [6, 7], we increase the number of cases nearly fourfold and omit features related to diffusion MRI. Additionally, the HcF network proposed in this work is narrower and deeper than that proposed in earlier work [6]. Model simplicity in this work may have resulted after data stratification. Whilst the cases included in this study span across a total of 5 years with differences in scanner and surgical techniques, our data are from only one centre and electrode manufacturer (Ad-Tech), which may limit the generalisability of our work. Also, although we transform electrodes into MNI space using an affine transformation, we highlight that anatomical structures in this space still differ across patients. Therefore, we investigate the predictive capabilities of the proposed models in patient-specific cases given the inputs.

In this work, we formulate the learning problem based on the brain structures corresponding to the entry and target points of electrodes rather than a single group with all electrodes. Although cases are balanced across groups, the number of electrodes differs with $\approx 90$ electrodes in ***ifog***, and ***apcg*** groups, followed by 117 in ***po***, $\approx 155$ in ***sfg***, and ***mfg***, and 242 in the ***tg*** groups. This is important to consider since predicting electrode trajectories in po regions are more difficult to predict.

Although we were unable to confirm whether **lu** or $\hat{\mathbf{eb }}$ labels were better predictors of electrode bending from the tenfold model cross-validation, we confirmed that **lu** has a higher predictive performance than $\hat{\mathbf{eb }}$ when computing trajectories via inference. Our results indicate that the HcF approach was outperformed by an E2E approach that only uses electrode direction and a 3D image window, particularly when using CWD images for $\mathbf{lu}$. This means that for ***sfg***, ***mfg***, ***ifog***, and ***tg*** data groups, a mask of the main brain structures is as good as, and sometimes better than, using the T1w intensities. However, for ***apcg*** and ***po*** regions, T1w has a greater predictive performance, indicating perhaps the T1w intensities provide additional information for these electrodes. This suggests that deviation from rigid trajectories can be mostly explained at interfaces/boundaries between brain structures, which we hypothesise is related to the different biomechanical properties of these tissues.

When inferring trajectories iteratively for best performing models, bending most often occurred at the interface between the cortex and white matter. However, when bending occurred, our approach was sometimes unable to predict bending observed in deeper points along the trajectory, resulting in trajectories that were overestimated. This has implications to our approach since errors accumulate as trajectory inference is an iterative process in our current formulation. Rather than predicting iteratively local displacement at interpolated points, predicting the entire trajectory at once may result in more accurate predictions. Folds 1 and 2 had reduced performance compared to the other eightfolds when inferring trajectories. Whilst investigating this, we noticed that model cross-validation of CWD **lu** is also affected by folds 1 and 2, and $MSE_i$ decreases considerably if this twofold is discarded for ***sfg***, ***mfg***, ***ifog***, ***tg***, ***apcg***, and ***po*** to 0.0007(0.001), 0.0303(0.046), 0.0003(0.0005), 0.0005(0.0009), 0.0006(0.0015), and 0.0015(0.002), respectively (see Table 1; last column). However, it is still unclear why these folds had reduced performance. Furthermore, whilst the $MSE_i$=1 mm threshold was used as a reference to compare best performing models, $MSE_i$ of folds 3-10 in Fig. 5 suggests that this threshold could be reduced to better capture the quality of predictions.

## Conclusions and future work

We presented two machine learning approaches based on handcrafted features and convolutional neural networks for predicting deviations of implanted SEEG electrodes from rigid trajectories in MNI space. Models were trained and tested using tenfold cross-validation on a dataset of 86 retrospective SEEG implantation cases from a single centre. We found that a streamlined end-to-end (E2E) approach that predicts local displacement and that uses an image containing only the masks of cortex, white, and deep grey matter (CWD) along the electrode trajectory showed the best performance, followed closely by a similar approach that uses a T1-weighted MRI image. However, using global displacement as a label for regression and accounting for spatiotemporal predictions may be an alternative to local displacements that remains to be investigated. Further studies are required to assess the generalisability of pre-trained models to other manufacturers and surgical centres. Furthermore, our work could be extended to other stereotactic interventions, such as deep brain stimulation, where brain shift may play a bigger role in electrode deflections. More importantly, further research will investigate the incorporation of electrode bending prediction and uncertainty quantification into planning algorithms. With the advent of new technologies for neuroprosthesis [12] and even brain computer interfaces [10], the work proposed here is a step forward towards more accurate patient-specific predicted implantations.

## Supplementary Information

Below is the link to the electronic supplementary material.

Supplementary material 1 (pdf 5515 KB)

## Footnotes

1 Source code: URL https://github.com/agranadoseu/SEEG-Electrode-Bending.
