# Supplementary material for: Patient-specific prediction of SEEG electrode bending for stereotactic neurosurgical planning
Source: Int J Comput Assist Radiol Surg. 2021 Mar 24;16(5):789–98. doi: 10.1007/s11548-021-02347-8 (PMC8134306; doi:10.1007/s11548-021-02347-8)
Supplement: Supplementary file 1 — Supplementary material 1 (pdf 5515 KB) [file 11548_2021_2347_MOESM1_ESM.pdf]

Noname manuscript No.
(will be inserted by the editor)

Patient-specific Prediction of SEEG Electrode Bending for Stereotactic Neurosurgical Planning
Supplemental Material

Alejandro Granados · Yuxuan Han · Oeslle Lucena · Vejay Vakharia · Roman Rodionov · Sjoerd B. Vos · Anna Miserocchi · Andrew W. McEvoy · John S. Duncan · Rachel Sparks · Sébastien Ourselin

Received: date / Accepted: date

Abstract *Purpose:* Electrode bending observed after stereotactic interventions is typically not accounted for in either computer-assisted planning algorithms, where straight trajectories are assumed, or in quality assessment, where only metrics related to entry and target points are reported. Our aim is to provide a fully automated and validated pipeline for the prediction of stereo-electroencephalography (SEEG) electrode bending. *Methods:* We transform electrodes of 86 cases into a common space and compare features-based and image-based neural networks on their ability to regress local displacement ($\mathbf{lu}$) or electrode bending ($\hat{\mathbf{eb}}$). Electrodes were stratified into six groups based on brain structures at the entry and target point. Models, both with and without Monte Carlo (MC) dropout, were trained and validated using 10-fold cross validation. *Results:* Image-based models outperformed features-based models for all groups, and models that predicted $\mathbf{lu}$ performed better than for $\hat{\mathbf{eb}}$. Image-based model prediction with MC dropout resulted in lower mean squared error (MSE) with improvements up to 12.9% ($\mathbf{lu}$) and 39.9% ($\hat{\mathbf{eb}}$), compared to no dropout. Using an image of brain tissue types (cortex, white and deep grey matter) resulted in similar, and sometimes better performance, compared to using a T1-weighted MRI when predicting $\mathbf{lu}$. When inferring trajectories of image-based models (brain tissue types), 86.9% of trajectories had an MSE$\leq$ 1 mm. *Conclusion:* An image-based approach regressing local displacement with an image of brain tissue types resulted in more accurate electrode bending predictions compared to other approaches, inputs, and out-

A. Granados, Y. Han, O. Lucena, R. Sparks, S. Ourselin
School of Biomedical Engineering & Imaging Sciences, King's College London, UK
https://www.kcl.ac.uk/bmeis
E-mail: alejandro.granados@kcl.ac.uk

V. Vakharia, R. Rodionov, S. B. Vos, A. Miserocchi, A. W. McEvoy, J. S. Duncan
National Hospital for Neurology and Neurosurgery, London, UK

puts. Future work will investigate the integration of electrode bending into planning and quality assessment algorithms.

Keywords Surgical planning · SEEG · Prediction of trajectory.

1 Data

Table 1 Electrode trajectories are stratified into six groups based on entry and target points. Anatomical regions are identified from the GIF parcellation obtained from the T1w.

		groups					
		**superior frontal gyrus (sfg)**	**middle frontal gyrus (mfg)**	**inferior frontal orbital gyrus (ifog)**	**temporal gyrus (tg)**	**anterior/posterior central gyrus (cg)**	**parietal occipital lobes (po)**
	**cases**	62	76	60	74	56	62
	**electrodes**	150	163	87	242	93	117
**entry point (EP)**	*... gyrus*	- superior frontal	- middle frontal	- lateral orbital<br>- opercular/triangular/orbiutal inferior frontal	- inferior/medial/superior temporal	- posterior central<br>- anterior	- angular<br>- supramarginal<br>- inferior/middle/superior occipital
	others	- supplementary motor cortex<br>- frontal pole		- frontal operculum	- temporal lobe		- superior parietal lobule<br>- occipital lobe
**target point (TP)**	*... gyrus*	- superior frontal<br>- anterior/middle cingulate<br>- anterior/medial/posterior orbital<br>- rectus	- anterior/middle cingulate<br>- anterior/medial/posterior/ lateral orbital<br>- superior frontal<br>- rectus	- anterior cingulate<br>- rectus<br>- medial orbital<br>- lateral orbital<br>- opercular/triangular inferior frontal	- fusiform<br>- lingual<br>- parahippocampal<br>- posterior cingulate<br>- traverse temporal<br>- inferior/medial temporal	- middle/posterior cingulate<br>- anterior medial segment	- middle/posterior cingulate<br>- fusiform<br>- lingual<br>- angular<br>- inferior occipital<br>-superior temporal
	... white matter	- cingulate<br>- frontal	- cerebral<br>- insula<br>- cingulate<br>- frontal	- cerebral<br>- frontal	- cerebral<br>- temporal<br>- insula<br>- frontal<br>- occipital<br>- periventricular	- cerebral<br>- insula<br>- cingulate<br>- frontal<br>- parietal	- cerebral<br>- cingulate<br>- parietal<br>- temporal<br>- occipital
	others	- supplementary motor cortex<br>- claustrum<br>- anterior insula<br>- medial frontal cortex<br>- sfg medial segment	- supplementary motor cortex<br>- lateral ventricle<br>- corpus callosum<br>- claustrum<br>- anterior insula<br>- central operculum<br>- medial frontal cortex<br>- sfg medial segment<br>- frontal operculum	- frontal operculum<br>- putamen<br>- corpus callosum<br>- claustrum<br>- anterior insula<br>- central operculum<br>- medial frontal cortex	- amygdala<br>- hippocampus<br>- lateral ventricle<br>- ventral dc<br>- entorhinal<br>- claustrum<br>- planun polare<br>- temporal lobe	- corpus callosum<br>- claustrum<br>- anterior/posterior insula<br>- central operculum<br>- frontal operculum<br>- supplementary motor cortex	- corpus callosum<br>- precuneus<br>- posterior insula<br>- parietal operculum<br>- amygdala<br>- lateral ventricle<br>- calcarine cortex<br>- cuneus<br>- superior parietal lobule

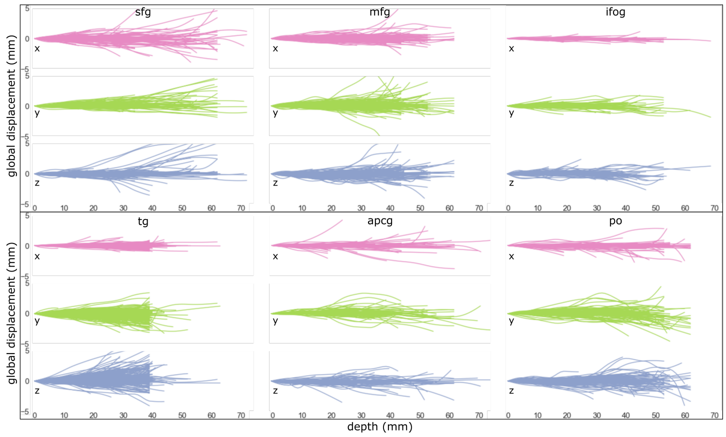

Fig. 1 Electrode deviations from straight trajectories as observed from electrodes identified from post-operative CT. Deviations from rigid trajectories in mm (global displacement) for x (lateral displacement; pink), y (upward/downward displacement; green), and z (anterior/posterior; blue) axis in MNI space are plot for each anatomical group. Each line represents a component of an electrode trajectory up to an implanted depth.

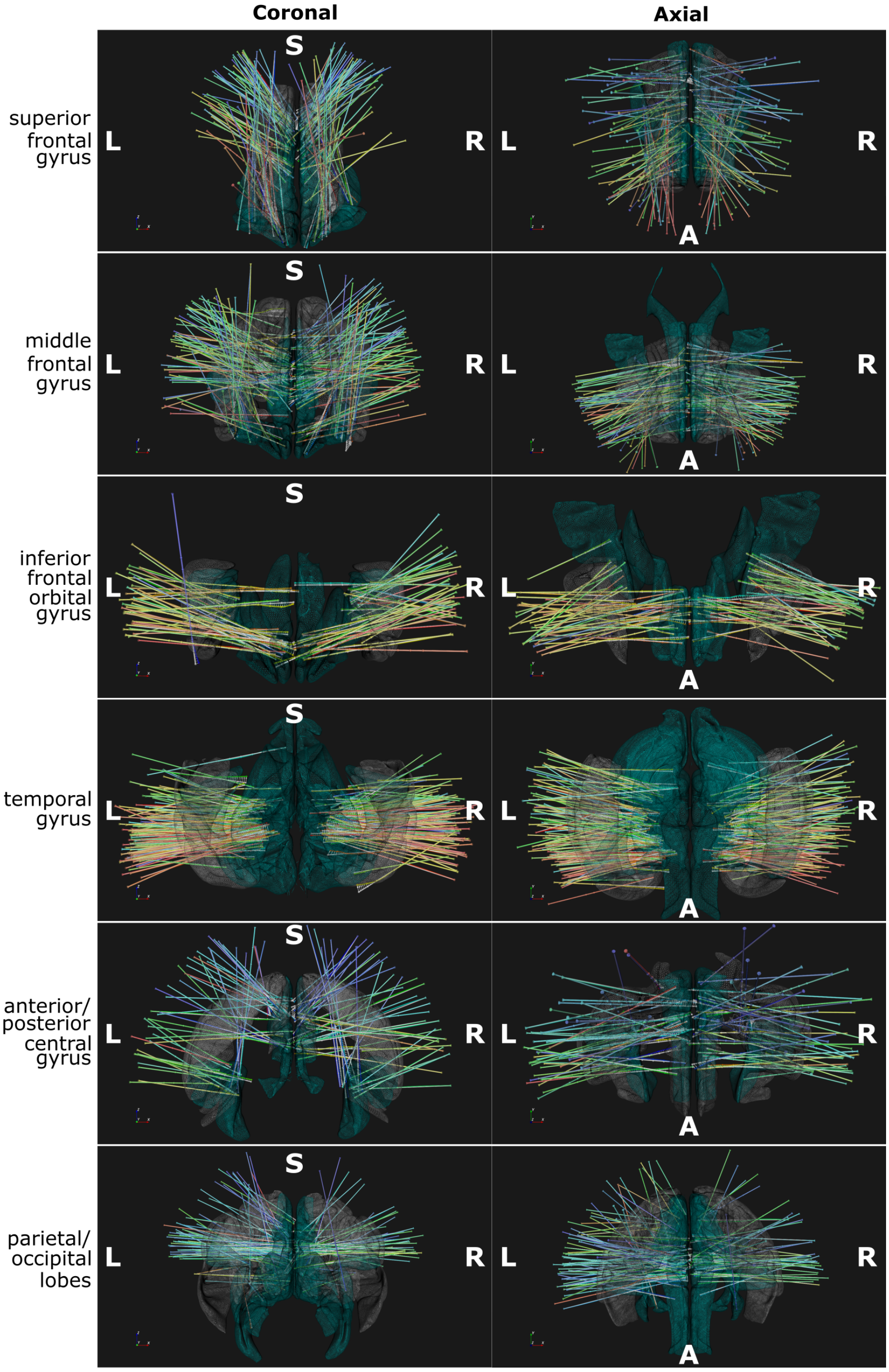

Fig. 2 Visualisation of the SEEG electrode trajectories dataset used in our study. Coronal (left) and axial (right) views of electrode trajectories stratified by anatomical regions (see Table 1 in main manuscript). Regions related to entry point (white wireframe mesh) and target point (green wireframe mesh) are shown.

Table 2 Hand-crafted features and labels. Features are grouped into five categories related to the electrode, amount of bending, T1w, GIF, and collision with surface meshes. Features/labels can be scalar (S), continuous (C), or 3D vector (V) types for the entire trajectory (*top*) or at interpolated points along the trajectory (*bottom*). * indicates normalisation via z-score.

	**Features**					**Labels**
	electrode	***bending***	***T1w***	***GIF***	***collision***	
entire trajectory	- number of contacts (S)	- bolt direction (V)	- intracranial depth* (S)	- region at EP (C)<br>- region at TP (C)<br>- white matter ratio* (S)	- position/normal crossing scalp/cortex (V)<br>- angle wrt scalp/cortex (S)	
at interpolated points	- stylet (C)<br>- position of implantation/ rigid (V)	- electrode direction (V)<br>- local/global bending* (V)<br>- curvature* (S)<br>- velocity* (S/V)<br>- acceleration* (S/V)	- voxel indices (V)<br>- T1w intensity (S)<br>- depth* (S)	- region (C)<br>- regions traversed* (S)<br>- cortex/white/deep (CWD) (C)<br>- cortex/white/deep distance traversed* (S)<br>- segment length* (S)<br>- segment depth* (S)<br>- segment white matter ratio* (S)	- distance to falx cerebri plane* (S)<br>- position/normal crossing white/deep matter (V)<br>- angle wrt white/deep matter* (S)	- local displacement (V)<br><br>- electrode bending direction (V)

2 Training time

Table 3 Mean training time (hh:mm:ss) of one fold of hand-crafted (HcF) and end-to-end (E2E) models when regressing local displacement (**lu**) and electrode bending ($\hat{\mathbf{eb}}$) labels across anatomical groups. The fastest model of each group is highlighted in bold.

		**HcF**	**E2E**		
**Label**	**Group**	-	*MRI*	*GIF*	*CWD*
lu	sfg	00:05:40	**00:04:40**	00:07:07	00:07:13
	mfg	00:06:07	00:06:40	00:08:07	**00:05:40**
	ifog	**00:03:33**	00:03:40	00:03:47	00:03:40
	tg	00:12:00	**00:07:00**	00:07:27	00:08:07
	apcg	00:04:27	00:04:00	**00:03:40**	00:03:47
	po	**00:04:13**	00:06:20	00:04:20	00:04:20
$\hat{eb}$	sfg	00:15:27	00:05:53	00:04:47	**00:04:40**
	mfg	00:17:27	00:06:20	**00:04:07**	00:04:27
	ifog	00:10:00	00:03:47	**00:02:27**	00:02:33
	tg	00:15:20	00:18:13	00:09:20	**00:05:47**
	apcg	00:07:13	00:03:40	00:02:53	**00:02:47**
	po	00:11:47	00:04:13	**00:03:47**	**00:03:47**

3 Inference

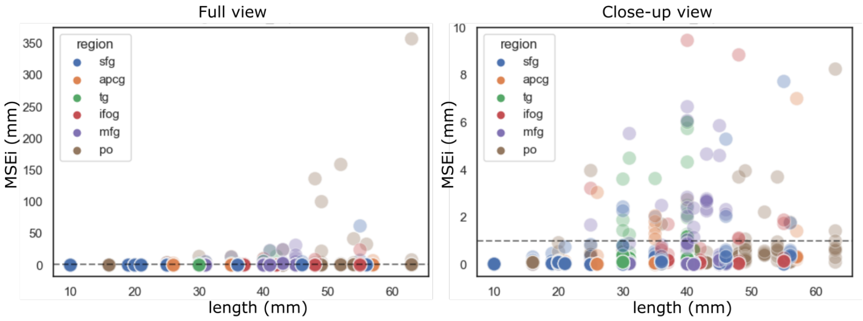

	sfg	mfg	ifog	tg	apcg	po
Pearson's r	0.18	0.16	0.21	0.14	0.18	0.18
p-value	0.02	0.05	0.08	0.03	0.13	0.02

Fig. 3 Correlation between electrode length and MSE_i across anatomical groups. *Top:* Full (left) and close-up (right) views of a scatter plot of electrode length and MSE of trajectory prediction versus ground truth (i.e. MSE_i). A horizontal dashed line indicates MSE_i values at 1 mm. *Bottom:* Table of Person's correlation coefficient r and corresponding p-values.
